# Fever and Ague

**Published:** 1875-05

**Authors:** 


### FEVER AND AGUE

Is more prevalent during the spring and fall than at other seasons; the reason is that changes of temperature are then more sudden and more frequent. There are persons who have lived many years in fever and ague districts without having had the disease. With proper care and attention, all might avoid it. An observance of these simple rules would generally ward off the disease.

Avoid exposure to the damp air of the early morning and the early evening, except when exercising, and then do not remain in the open air to cool off. Avoid great fatigue; sleep eight hours out of twenty-four. Be sure that the water used for drinking and cooking is perfectly pure. Wear flannel underclothing at all seasons. Keep the feet dry and warm.

To cure fever and ague take 12 grains of quinine, AT ONE DOSE, about an hour before the chill is expected. Just one week from that hour take another 12 GRAINS of quinine. The disease will seldom return. This is the dose for an adult. Children should take smaller doses, according to age. The reason that decided doses of quinine cure fever and ague, seems to be, that the disease receives a shock which breaks it. Small doses of quinine only hold it in check during the time the medicine is being regularly taken; as soon as it is suspended the disease usually returns. Hence the popular notion that the quinine only "feeds" the disease. The fault is not with the medicine, but in the manner of administering it. While we do not believe in encouraging the employment of medicine, we are bound to say that quinine, heroically administered, has proved the only "dead-shot" for fever and ague in our practice.

---

IF a man desires to live within his means, and is resolute in his purpose not to appear more than he really is, let him be applauded. There is something fresh and invigorating in such an example, and we should honor and uphold such a plan with all the energy in our power. But how difficult to stem the direction of culture in our best circles where approbativeness is nursed and tickled into excessive growth in childhood, and consequently bears its fruitage of vanity, display, and supercilious obedience to conventionalities in mature life. The extravagance of the development may, in time, bring about a reform.

But just now the world is crazy for show. There is not one, perhaps, in a thousand, who dares fall back on his real, simple self for power to get through the world and exact enjoyment as he goes along. There is no end to the apeing, the mimicry, the false airs and the superficial acts. It requires rare courage to live up to one's enlightened convictons in these days. Unless one consents to join in the general cheat, there is no room for him among the great pretenders. May we not indulge the hope that by and by the intelligent classes will consent to frown down this demoralizing, artificial, unnatural life, and rise to a higher and purer system? It takes time, of course, but everything convinces us that we are doing vastly better than did those who have lived before us. So we hope for great things by and by.

